# Best Practices Guidelines for the Engagement of People With Lived Experience and Family Members in Mental Health and Substance Use Health Research: A Modified Delphi Consensus Study

**DOI:** 10.1111/hex.70152

**Published:** 2025-01-20

**Authors:** Lisa D. Hawke, Wuraola Dada‐Phillips, Hajar Seiyad, Josh Orson, Lianne Goldsmith, Susan Conway, Adam Jordan, Natasha Y. Sheikhan, Melissa Hiebert, Sean Kidd, Kerry Kuluski

**Affiliations:** ^1^ CAMH Education Research University of Toronto Department of Psychiatry Toronto Canada; ^2^ CAMH Education Research Toronto Canada; ^3^ CAMH Patient and Family Engagement Core Toronto Canada; ^4^ CAMH Education Research University of Toronto Institute for Health Policy, Management and Evaluation Toronto Canada; ^5^ CAMH Slaight Family Centre for Youth in Transition University of Toronto Department of Psychiatry Toronto Canada; ^6^ University of Toronto Institute for Health Policy, Management and Evaluation Trillium Health Partners Institute for Better Health Toronto Canada

**Keywords:** best practice guidelines, engagement in research, mental health, patient engagement, people with lived experience, substance use

## Abstract

**Introduction:**

People with lived experience of mental health and/or substance use conditions and their families (PWLE) are increasingly engaged in research, yet rigorous guidelines for engagement are lacking. This study aims to co‐design best practice guidelines to support the authentic, meaningful engagement of PWLE in mental health and/or substance use health research.

**Methods:**

A multi‐panel modified Delphi study was conducted with 61 expert panelists (35 PWLE and family members, 26 researchers/research support staff from across Canada). Participants rated 56 recommendations for importance and clarity. Consensus was defined as ≥ 70% of participants rating items at 6 or 7 on a 7‐point Likert scale (‘very important’ or ‘essential’). Qualitative feedback was analysed using content analysis to identify new items and reviewed for improvements in item clarity. After each round, items not meeting the established threshold of importance were removed. Items with low clarity scores were reworded. A PWLE advisory panel was actively involved throughout the study's design, implementation, interpretation, and reporting, ensuring that the perspectives of people with lived experience were integrated throughout the research process.

**Results:**

Three Delphi Rounds were conducted. In Round 1, importance ratings ranged from 51.7% to 96.7% of participants ranking the items above the established threshold (average 80.1%), with clarity ratings ranging from 39.3% to 86.9% (average 70.7%) and an average importance coefficient of variation (CV) of 0.16. Four items were deleted, two new items were added and fifty‐five items were revised. In Round 2, 60 (98.4%) participants responded. Importance ratings ranged from 57.6% to 96.7% (average 80.2%; average CV = 0.20). Clarity ratings ranged from 50.9% to 93.2% (average 77.9%). Five items were deleted and eleven revised. In Round 3, 60 (98.4%) participants provided importance ratings ranging from 66.7% to 98.3% (average 80.8%; average CV = 0.20), and clarity ratings ranging from 63.3% to 94.9% (average 81.1%). Three items were deleted and nine were revised. Forty‐four final best practices are proposed.

**Conclusion:**

These co‐developed best practice guidelines offer recommendations for meaningful PWLE engagement in mental health and/or substance use health research. By following these guidelines, research teams can ensure that PWLE contributions are genuinely valued and effectively integrated, ultimately enhancing the quality and impact of the research and fostering authentic collaboration.

**Patient and public involvement:**

People with lived experience were engaged throughout the project as key team members, from a patient‐oriented research perspective. They are also co‐authors on this manuscript.

## 1 Introduction

The engagement of people with lived experience of mental health and/or substance use conditions and their family members (PWLE) in research about them and their needs is an increasing priority in health research [1, 2, 3]. Extending beyond a paradigm in which PWLE are subjects to be studied, patient‐oriented research practices call for them to be members of the research team, serving in the role of advisors, collaborators, co‐researchers, or analogous roles. PWLE can advise, collaborate, co‐design, and/or lead many aspects of research, such as setting research priorities, conducting data collection, analysing and interpreting data, and conducting knowledge translation [3, 4]. PWLE engagement is productive across a wide range of study designs and research topics [4, 5, 6]. Some view PWLE engagement as a critical ethical imperative and anti‐oppressive practice in light of past and current inequities in research and clinical practice that have negatively affected the people who are studied and treated [7, 8]. PWLE engagement is guided by pragmatism as a fundamental value and research paradigm, whereby experiential knowledge is highly valued alongside research evidence [9].

While PWLE engagement has been happening to a degree in mental health and/or substance use health research for years, it has become more mainstream in recent years, in a research climate that moves at a rapid pace and in which engagement has emerged as a growing priority [2]. Note that mental health and substance use are highly comorbid and they are increasingly being addressed together in research and clinical care [10]. A scoping review of the impacts of engagement suggests that engaging PWLE in research can bring many benefits to the research, to the individuals engaged, and to researchers [2]. That review suggests that when PWLE are engaged in research, the resulting research is more likely to be aligned with the needs and priorities of the population of interest, becoming more likely to be feasible, easily adopted, implemented, and sustainable. However, it also reveals barriers, making PWLE engagement a challenging process for many research teams and one that requires specific attention to optimize practice.

Various frameworks and materials are available to support PWLE engagement in research, such as the Health Quality Ontario Patient Engagement Framework [11], the Patient Engagement in Research conceptual framework and accompanying psychometric scale [12, 13], the UK Public Involvement Standards [14], and various online resources and tools offered by funding bodies and other groups, such as the Patient‐Centred Outcomes Research Institute (PCORI) [15], Wellcome Trust [16], and the Patient Engagement Resource Centre [17]. Hart's Ladder and the IAP2 frameworks are frequently cited descriptors of levels of engagement [18, 19]. A paucity of guidance is available specific to family member engagement. In a 2018 publication [20], a Delphi study was conducted to identify attributes of successful PWLE engagement in research. However, that study was not specific to mental health and/or substance use. In addition, the resulting recommendations were largely broad and values‐based, without addressing the practical day‐to‐day management of engagement. Some guidance exists in the grey literature for engagement more broadly [21], but not specific to engagement in mental health and substance use health research. Together, these resources provide some guidance to researchers; however, most of the resources available are not specific to mental health and/or substance use, leaving gaps with regard to the particular needs of PWLE in the mental health and substance use health field. Indeed, concrete, step‐by‐step guidance that reflects the realities of engagement across contexts in this field of research is generally lacking.

Engagement in mental health and substance use health research is different from engagement in physical health research for a number of reasons. Notably, past and present injustices and oppression specific to treatment, such as paternalistic care cultures and a lack of recovery‐oriented care and hope‐based perspectives, make mental health and substance use health research a priority area for engagement [7, 22, 23]. It is well documented that stigma toward mental illness and substance use exists within healthcare fields [24, 25], which is a barrier that affects the engagement process [26]. An ‘us versus them’ academic culture, skepticism, and mistrust in research, coupled by past and present inequities in research and care have emerged as additional challenges to overcome [2, 27]. Issues related to cultural sensitivity and trauma‐informed approaches are also particularly important in mental health and substance use health research, given the multifaceted relationships between social determinants of health, trauma, and mental health and/or substance use conditions. The lack of clinically relevant biomarkers for most mental illnesses differs mental illness from physical health, emphasizing the intersection between mental illness, emotions, opinions, and social location. While representativeness is a challenge across health disciplines, it is especially important in this area of research, since mental health and substance use health intersect with social determinants of mental health [28] that may sometimes affect the way PWLE engage with research. Together, this myriad of factors makes engagement in this area of research unique and calls for practical, domain‐specific best practice guidelines.

Despite the expanding guidance provided by the literature, the evidence base guiding the engagement of PWLE in mental health and substance use health research remains limited. Research is required to clarify research and practice components important to the engagement of PWLE in research. Researchers who are conducting mental health and substance use health research engaging PWLE and the PWLE they engage are ideally positioned to guide the advancement of engaged research processes and methodologies.

### 1.1 Objective

This modified Delphi consensus study aimed to develop best practice guidelines for PWLE engagement in mental health and substance use health research, including a spectrum of guidance ranging from broad values‐based overarching principles to specific, concrete practice recommendations.

## 2 Method

This project is a multi‐panel ‘modified’ virtual Delphi consensus study. In a modified Delphi, pre‐existing evidence and consultations are used to draft initial Delphi items, which are then submitted to rating by expert panels of study participants [29]. The Patient Engagement in Research (PEIR) conceptual framework guided the lived experience engagement in this project [12]. A guideline development team consisted of the research lead, research analyst, and the PWLE Advisory Group (three people with lived experience and two family members), who met on a regular basis to guide this work. Other team members were also consulted as needed. Leveraging our existing work, including two scoping reviews [30, 31], we drafted an item map and an initial set of items for use in the first round of the Delphi consultation; in using the ‘modified’ approach, we were able to use our existing literature reviews and the expertise of our PLWE advisory group, while providing expert panelists with initial items to reflect upon. The Delphi study then took place in accordance with accepted Delphi methodology [29]. Results are reported in accordance with Delphi reporting guidelines [32], while lived experience engagement is reported using the GRIPP‐2 checklist (Table 1) [33]. We followed paradigms of constructivism and pragmatism [9, 34], in line with Delphi methodology and PWLE engagement.

**Table 1:** Guidance for Reporting Involvement of Patients and the Public (GRIPP2) reporting checklist for lived experience engagement in research.

Section and topic	Description
1. Aim	This study aimed to cocreate best practice guidelines for the engagement of PWLE, together with PWLE, through both deliberative and consultative engagement [44].
2. Methods	Two PWLE joined the grant development team and contributed to the initial protocol development. Upon funding, a PWLE Advisory Group was established, made up of the original 2 PWLE from the grant application, plus one more PWLE and 2 family members. They met a total of 13 times from project launch to the manuscript submission process. They deliberated on study design, guideline ideas, recruitment ideas, data analysis, interpretation, and reporting.
3. Study results	The PWLE helped codevelop the initial items. They edited and improved the study flyers to be more engaging to potential participants. They helped edit the content and design of the participant‐friendly videos developed for each round to orient participants to the Delphi process. The PWLE team met after each Delphi round as the expert advisory group to review the round's results and reword recommendations. They discussed the content of this table and co‐developed the content within it. They co‐authored the current manuscript and the best practice guidelines document.
4. Discussion and conclusions	This was an effective and efficient advisory group that contributed meaningfully to all stages of the study. Minor challenges were encountered. The task of refining the items in simple terms based on participant feedback was challenging for the team. Interpreting the qualitative comments without talking to the participants directly was another challenge, which was mitigated through open dialogue and continually returning to the data. Advisors also expressed that it was challenging to remember to be objective about the results even when they did not agree with them. We navigated these challenges by taking as many suggestions as we were able to take, while continuing to emphasize the need for simplicity, clarity, and objectivity while referring back to the qualitative comments. Advisors described that balancing meeting attendance with life challenges was also a minor challenge they encountered. We made accommodations by being flexible with meeting schedules and attendance. We further provided regular updates on the way their advice was used, ensuring continued participation and engagement.
5. Reflections/critical perspective	The PWLE Advisory Group reflected on our engagement approach. They appreciated the regular meeting reminders, which allowed consistent communication. Regarding study design, they suggested that incorporating qualitative interviews could have provided a deeper understanding of participants' feedback during the survey rounds. The study allowed them to reflect on their own engagement work with other research teams and critically assess aspects of our recommendations that were missing in those studies. Importantly, they expressed that we successfully created a supportive environment where they felt comfortable sharing their perspectives. They also expressed that they felt respected as equal contributors and noted that our focus on leveraging their skills demonstrated genuine respect for their contributions. For them, authentic and meaningful engagement meant that their contributions were genuinely valued, and that tangible changes were made based on their feedback.

### 2.1 Participants and Recruitment

**Figure 1:**
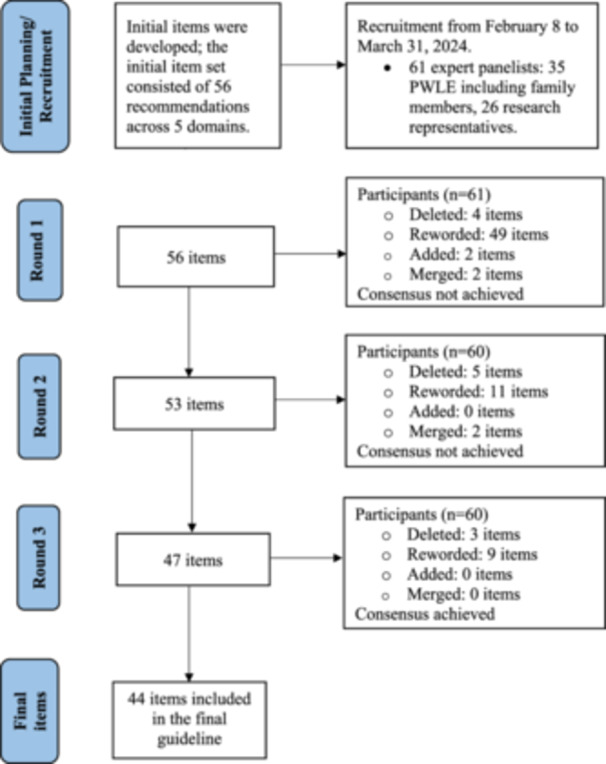
Flow diagram of our Delphi consensus study process.

An overview of our Delphi study process is shown in Figure 1. PWLE and researchers were eligible to participate if they were 16 years of age or older, able to speak English, and residing in Canada. All participants had to have experience with at least one PWLE‐engaged study in mental health and/or substance use. A sample of 61 participating expert panelists were recruited from across Canada (35 PWLE including family members, 26 mental health/substance use researchers). This sample size exceeds the recommended minimum of 20 participants per panel for Delphi studies [35]. Among the PWLE sample, 20 identified primarily as people with personal lived experience and 15 identified primarily as family members. Expert panelists were recruited by circulating a project flyer throughout our networks using email and social media. This included circulation to the Research Advisory Network of the Centre for Addiction and Mental Health (CAMH), via project newsletters and social media feeds at the Strategy for Patient‐Oriented Research (SPOR) Evidence Alliance, the Passerelle National Training Entity in Patient Engagement, and the Ontario SPOR Support Unit. Flyers were also sent to the network of provincial SPOR bodies and relevant patient‐oriented researchers across Canada.

### 2.2 Procedure

Interested potential participants connected with the research analyst for more information. The analyst determined eligibility, described the study, collected electronically signed informed consent, and sent a link to a basic demographic form hosted on REDCap software [36]. Participants were then considered expert panelists and proceeded with the first round of the Delphi study via a unique REDCap link. First, they had the option to view a co‐designed video tutorial that described the Delphi process in lay language, as recommended in the literature [35]. They then completed the online Delphi survey. The survey was then closed and analyses were conducted. The same procedure was repeated for subsequent Delphi rounds. Each round had a unique video orienting participants to that round. Round 1 was open for recruitment from February 8 to March 31, 2024. Round 2 data collection occurred from May 2 to 20, 2024 and Round 3, from June 14 to July 12, 2024. Items were iteratively refined through three rounds of consultation. Expert panelists received a $50 e‐gift card for each Delphi round.

### 2.3 Survey

The initial Delphi survey items were drafted by members of the project team, including the investigators and the PWLE advisory group, and based on the scoping reviews underpinning this work [30, 31]. The item set consisted of 56 draft recommendations, each with an open‐ended question for qualitative feedback, and one final open‐ended question for participants to suggest additional items. The items were divided into a five‐category content map by the team based on the stages of research including a section on (1) initial reflections and underlying values (9 items), (2) planning to engage people with lived experience (13 items), (3) onboarding people with lived experience (6 items), (4) working with people with lived experience (20 items), and (5) evaluating and reporting on engagement (8 items). Participants rated each recommendation regarding (1) the importance and (2) clarity, on a 7‐point Likert scale ranging from ‘not at all important/clear’ to ‘essential/completely clear’ [37].

#### 2.3.1 Analyses

Statistical analyses were conducted using IBM SPSS Statistics, version 27, and Microsoft Excel [38, 39]. Qualitative content analysis was conducted using NVivo version 12 [40]. The two panels (PWLE, researchers) were analysed first separately, then synthesized. Importance and clarity ratings were descriptively synthesized. We conducted subgroup analyses, from an equity standpoint, on subsamples (youth, gender, ethnically diverse participants), reflecting the constructivist nature of Delphi studies [34] and to collect minority views. We notably inspected proportions of respondents endorsing each item at each anchor level on the 7‐point Likert scale. Items for which at least 70% of participants rated importance as a 6 or 7 (i.e., ‘very important’ or ‘essential’) on the Likert scale in the primary analyses or subgroup analyses were retained for the next round [37]. Percentages were prorated out of the number of valid answers in the case of missing data, which were minimal. Clarity ratings were analysed in conjunction with pragmatic qualitative comments, many of which referred to item clarity. Items with low clarity scores and/or relevant qualitative comments regarding clarity were reworded through discussion with the PWLE advisory group to enhance understanding. In Round 1, responses to an open‐ended item proposing additional potential items were analyzed using content analysis by one coder for the generation of new items [41]. We calculated the coefficient of variation (CV) for each item to determine item stability and guide us with determining when to cease data collection [35]. CVs were interpreted in conjunction with the proportions and the qualitative feedback. As an alternate measure of stability, we calculated the Pearson correlation coefficient (*r*) between the mean CVs in Rounds 1 and 2, and in Rounds 2 and 3. We also calculated the correlation between mean item scores in Rounds 1 and 2, and in Rounds 2 and 3, which we interpreted using standard interpretations [42].

#### 2.3.2 Context

This study was led by a scientist specializing in PWLE engagement in mental health and substance use health research, supported by a team of co‐investigators, knowledge users, PWLE, and a research analyst, all with expertise in this area of work. It was conducted out of an urban tertiary care mental health and substance use hospital with a strong established PWLE engagement infrastructure in place. The project was funded via a Patient‐Oriented Research Priority Announcement by the Canadian Institutes of Health Research awarded to the first author. The study was approved by the Research Ethics Board of the Centre for Addiction and Mental Health.

## 3 Results

The demographic characteristics of participants are presented in Table 2. Many of the researcher participants were young professionals, with a somewhat older PWLE sample. A majority of participants were women in both groups, although some men and transgender/nonbinary individuals were represented. The researcher group was racialized in the majority, while the PWLE group was white as a majority. The majority of both groups was recruited from the province of Ontario, although nearly a quarter of the sample was from outside of Ontario. The researchers had a career focus on children (*n* = 3, 11.5%), youth (*n* = 14, 53.8%), adults (*n* = 20, 76.9%) and geriatrics (*n* = 5, 19.2%), while the PWLE were also engaged on projects across the lifespan. Researchers came from a wide range of disciplines, including psychiatry (*n* = 2, 7.7%), psychology (*n* = 6, 26.1%), social work (*n* = 2, 7.7%), public health (*n* = 5, 19.2%), nursing (*n* = 3, 11.5%), and other areas of work. Three rounds of Delphi consultations were conducted. The results of each round are presented in Table 3. The final Best Practice Guideline is provided in the Appendix.

**Table 2:** Sociodemographic characteristics of study participants.

Sociodemographic characteristics		PWLE Participants *n* = 35		Researcher Participants *n* = 26	
		*n*	%	*n*	%
Age	16–29	7	20.0	13	50.0
	30–59	20	57.1	9	34.6
	60+	8	22.9	2	7.7
Gender	Man	5	14.3	2	7.7
	Woman	27	77.1	22	84.6
	Transgender or nonbinary	3	8.6	2	7.7
Background	White	20	57.1	9	34.6
	Black	2	5.7	0	0.0
	East and South East Asian	2	5.7	4	15.4
	South Asian	8	22.9	6	23.1
	Middle Eastern	0	0.0	2	7.7
	Multiple identities	2	5.7	2	7.7
	Another ethnicity	0	0.0	2	7.7
	Missing	1	2.9	1	3.8
Province	Ontario	27	77.1	20	76.9
	Outside of Ontario	8	22.9	6	23.1
Highest level of schooling	High school or less	2	5.7	0	0.0
	Some postsecondary	10	28.6	1	3.8
	Graduated from college or university	10	28.6	5	19.2
	Post‐graduate degree	13	37.1	20	76.9
Age demographic of research experience	Child	5	14.3	3	11.5
	Youth	15	42.9	14	53.8
	Adult	23	65.7	20	76.9
	Geriatrics	7	20.0	5	19.2
PWLE Engaged	People with personal lived experience	n/a	n/a	25	96.2
	Family members	n/a	n/a	17	65.4
Stage of engagement experience	Early project planning (e.g., priority setting)	21	60.0	17	65.4
	Project design (e.g., selecting measures…)	16	45.7	22	84.6
	Project operations (e.g., recruiting)	17	48.6	21	80.8
	Data analysis and interpretation	17	48.6	21	80.8
	Lay knowledge translation	17	48.6	21	80.8
	Academic knowledge translation	8	22.9	18	69.2

**Table 3:** Proportion of participants endorsing items at 6 or 7 on the 7‐point Likert scale, by Delphi Round.

No.	Item construct	Round 1		Round 2		Round 3	
		Importance	Clarity	Importance	Clarity	Importance	Clarity
**A. Initial reflections and underlying values**							
1	Reasons for engaging	88.5%	39.3%	93.3%	60.0%	90.0%	81.7%
2	Healthcare system interaction^a^	80.3%	55.7%	71.2%	69.0%	66.7%	70.0%
3	Inequities, barriers^a^	86.9%	60.7%	88.3%	68.3%	81.7%	69.5%
4	Equal value	86.9%	80.0%	81.4%	81.0%	83.3%	83.1%
5	Subject‐matter experts	96.7%	86.9%	96.7%	93.2%	91.5%	90.0%
6	Identify novel factors	90.2%	72.1%	85.0%	86.4%	83.3%	83.3%
7	Diversity of experiences	95.0%	86.7%	91.7%	87.9%	96.7%	93.3%
8	Intersectionalities	68.3%^b^	54.2%	57.6%	76.3%	—	—
9	Unique insights	80.3%	47.5%	80.0%	76.3%	73.3%	76.7%
57	Family‐specific engagement	—	—	60.0%	61.0%	—	—
**B. Planning to engage PWLE**							
10	Engagement professional	52.5%	58.3%	—	—	—	—
11	Engagement ally	71.7%	75.0%	61.7%	62.7%	—	—
12	Research team training	88.1%	80.0%	93.3%	91.5%	93.3%	90.0%
13	Priority population	74.6%	61.4%	75.0%	58.3%	75.0%	74.6%
14	Exclusions	78.7%	68.9%	81.7%	81.7%	83.3%	78.3%
15	Where to find members	72.1%	55.7%	76.7%	66.7%	75.0%	68.3%
16	Recruitment materials	88.5%	78.3%	88.3%	80.0%	88.3%	88.3%
17	Engage early	72.1%	63.9%	88.3%	81.4%	83.3%	85.0%
18	Flexibility	86.9%	66.7%	91.7%	79.7%	88.3%	81.7%
19	Roles, responsibilities, tasks	95.1%	81.7%	91.5%	81.4%	89.8%	81.0%
20	Budget and fair pay	93.4%	86.9%	95.0%	89.8%	98.3%	93.2%
21	Power dynamics	83.3%	46.7%	78.3%	62.1%	81.7%	64.4%
22	Engagement meeting plan	76.7%	54.1%	78.0%	67.8%	71.7%	63.3%
58	Culturally relevant engagement	—	—	78.3%	72.4%	71.7%	68.3%
**C. Onboarding PWLE**							
23	PWLE training	75.4%	73.8%	75.0%	83.1%	78.3%	86.7%
24	Engage 2 + PWLE	60.7%^c^	61.7%	83.3%	79.7%	78.3%	85.0%
25	Rapport and accommodations	86.7%	73.3%	90.0%	82.8%	90.0%	85.0%
26	Introductions	75.0%	76.7%	73.3%	81.4%	78.3%	88.3%
27	Role clarity	85.3%	85.0%	—	—	_	_
28	Terms of reference document	75.4%	65.0%	81.7%	76.3%	75.0%	81.7%
**D. Working with PWLE**							
29	Privacy and confidentiality	90.2%	81.7%	90.0%	84.8%	83.3%	83.3%
30	Biases and assumptions	83.6%	63.9%	71.7%	71.4%	73.3%	71.7%
31	PWLE‐language	88.5%	77.1%	85.0%	84.8%	78.3%	83.1%
32	Mentorship and professional development	51.7%	71.2%	—	—	—	—
33	Flexibility about absences	88.5%	85.0%	78.3%	88.1%	81.7%	85.0%
34	Accommodate timing, scheduling	86.7%	78.3%	83.1%	91.4%	78.3%	90.0%
35	Be aware of triggers	93.4%	81.4%	93.3%	89.7%	88.1%	83.1%
36	Trauma‐informed engagement	86.9%	75.4%	88.3%	76.3%	85.0%	81.7%
37	Build rapport	90.2%	80.3%	93.3%	83.1%	88.3%	81.4%
38	Friendly environment	73.8%	70.5%	64.4%	72.9%	—	—
39	Clear, accessible materials	91.7%	83.1%	84.8%	84.8%	81.4%	78.0%
40	Send meeting materials	72.1%	78.7%	75.0%	86.4%	66.7%	81.4%
41	Plain language	78.7%	78.7%	75.0%	89.7%	71.7%	84.8%
42	Pre‐brief and debrief meetings	65.0%^d^	71.7%	73.3%	86.4%	66.7%	84.8%
43	Allow time to contribute	90.7%	72.1%	75.0%	61.0%	75.0%	71.2%
44	Consider suggestions	73.8%	72.1%	70.0%	83.1%	78.3%	83.3%
45	Communicate about changes	85.3%	82.0%	78.0%	86.4%	80.0%	86.7%
46	Discuss how to provide feedback	60.7%	70.5%	—	—	—	—
47	Tracking hours	85.3%	79.0%	83.3%	89.8%	79.7%	94.9%
48	Project closure meeting	88.5%	85.3%	89.8%	88.1%	88.3%	93.1%
**E. Evaluating and reporting on PWLE engagement**							
49	Consider why to evaluate	67.2%^e^	58.3%	61.7%	50.9%	—	—
50	Quantitative versus qualitative	56.7%	52.5%	—	—	—	—
51	Who and what to evaluate	78.3%	60.0%	66.7%^b^	50.9%	71.7%	63.3%
52	How engagement impacted	78.7%	74.6%	70.0%	74.1%	—	—
53	Coauthorship	77.1%	70.5%	71.2%	78.0%	71.7%	78.3%
54	Acknowledgements	86.7%	80.0%	81.7%	84.8%	76.7%	81.7%
55	Reporting clearly	63.9%^f^	62.3%	75.0%	72.9%	81.0%	74.6%
56	Feedback loop	85.0%	65.6%	88.1%	82.8%	85.0%	83.1%
**Average**		**80.1%**	**70.7%**	**80.2%**	**77.9%**	**80.8%**	**81.1%**

^a^ These items were moved to a different section in Round 2.

^b^ This item was retained because it was endorsed by >70% of researchers.

^c^ This item was retained because it was endorsed by >70% of family members and gender diverse participants.

^d^ This item was retained because it was endorsed by >70% of researchers and youth participants.

^e^ This item was retained because it was endorsed by >70% of PWLE and women.

^f^ This item was retained because it was endorsed by >70% of youth and racialized participants.

### 3.1 Round 1

A total of 61 participants completed Round 1. Across the item set, importance ratings ranged from 51.7% to 96.7% of participants ranking the items above the established threshold (average 80.1%), versus 39.3% to 86.9% (average 70.7%) for clarity. Twenty‐one items were below the 70% threshold for clarity; qualitative comments provided constructive feedback for the refinement of most items. The average mean was 6.14 and the average CV was 0.16, indicating a good degree of consensus among participants. According to the decision rule that CV ≥ 0.5 indicates a good degree of consensus [43], this suggests no need for an additional round based on the Round 1 results alone. However, based on the qualitative content analysis, lower clarity scores, and substantial feedback about rewording items, we determined that an additional round was needed.

Based on the sum of the Round 1 findings, the item set was revised. Due to low importance scores, we deleted items 10 (*have an engagement professional on the team*), 32 (*provide ongoing mentorship and professional development*), 46 (*discuss how to provide feedback*) and 50 (*consider qualitative and quantitative evaluations*). Forty‐nine items were reworded for clarity. Items 27 and 28 were merged into one reworded item (i.e., *be clear about roles, responsibilities, etc*. vs. *develop a terms of reference document*). Two items were revised in such a manner as they were changed to a different category in the content map, i.e., from *Initial reflections & underlying values*, to *Planning to engage* and *Onboarding* respectively. Lastly, two new items were generated, focusing on *family‐specific engagement considerations* and *culturally relevant engagement considerations*. A total of 53 items were put forward into Round 2.

### 3.2 Round 2

Nearly all participants completed Round 2 (60/61 = 98.4%). Importance ratings ranged from 57.6% to 96.7% (average 80.2%). Clarity ratings ranged from 50.9% to 93.2% (average 77.9%). The average mean was 6.06 and the average CV was 0.20. The *r* between Round 1 and Round 2 mean scores and CVs were 0.73 (strong) and 0.54 (moderate) respectively. Low clarity scores and qualitative comments suggested the need for further rewording of multiple items. A third round was therefore pursued.

Based on the Round 2 importance ratings, we deleted five items: items 8 (*recognize intersections between lived and professional experience*), 11 (*identify an engagement ally*), 38 (*establish a friendly environment*), 49 (*why to evaluate the PWLE engagement process*), and 57 (*consider family‐specific engagement considerations*). Importance and clarity scores and qualitative comments led us to merge two items (51 and 52; *consider what who and what to evaluate*, and *evaluate how engagement changed the research*). Based on clarity scores and qualitative comments, 11 items were reworded for clarity. A total of 47 items were moved forward to the third round of the Delphi.

### 3.3 Round 3

Retention for Round 3 was (60/61 = 98.4%). Participants provided importance ratings ranging from 66.7% to 98.3% (average 80.8%), and clarity ratings ranging from 63.3% to 94.9% (average 81.1%). The average mean score increased to 6.12 and the average CV remained at 0.20. Correlations for the average mean score and CV between Rounds 2 and 3 were 0.83 and 0.68, respectively. Increased *r* between CVs and between mean scores in Rounds 2 and 3 demonstrated a high degree of stability. Qualitative comments were limited and few items required revision. These results showed a consistent and good degree of consensus among expert panelists, suggesting no need for additional rounds.

Based on the sum of the Round 3 findings, three items were deleted: items 2 (*interactions with the healthcare system*), 40 (*send reading and meeting materials before meetings*) and 41 (*use plain language or visual prompts*). We reviewed and reworded nine items that did not meet the clarity threshold. A final set of 44 best practice guidelines are proposed. We reviewed the full final item set and moved seven items to a different subsection. The final guideline items are provided in Table 4. The fully developed Best Practice Guidelines with descriptive text are available online, in English and French, at https://www.camh.ca/en/professionals/professionals--projects/engaging-people-with-lived-experience-in-research.

**Table 4:** Final best practices guidelines for the engagement of PWLE in mental health and/or substance use health research.

**Initial reflections and underlying values**	
**1**	Consider why you want to engage PWLE and how their expertise can be included in your project.
**2**	Recognize that lived experience and research experience are different but equally valuable. Both can inform a research project.
**3**	Acknowledge that PWLE are experts who can inform research, make the research more relevant, and improve its quality.
**4**	Acknowledge that PWLE can identify factors influencing the research questions that you might have otherwise overlooked.
**5**	Recognize that everyone's experience is unique and that one PWLE cannot represent all people.
**6**	Appreciate the unique insights (about age, gender, mental health, etc.) that PWLE provide.
**Planning to engage people with lived experience**	
**7**	Plan to start engaging PWLE as early as possible in the research project.
**8**	Ensure that the team's scientists, trainees, and research staff have appropriate training on how to engage meaningfully with PWLE.
**9**	Identify what health conditions (e.g., mental health, physical health) or other characteristics (e.g., age, gender, ethnicity) you want the PWLE to have, based on the goals of the study.
**10**	Consider what populations you are intentionally or unintentionally excluding from your research and from your engagement initiative, why, and how that impacts your project.
**11**	Explore where you can recruit diverse PWLE who meet your criteria for engagement (e.g., age, ethnicity, mental health issue).
**12**	Develop materials to recruit PWLE who are representative of your priority population, with a clear indication of the role and commitment.
**13**	Plan to be flexible in the project and make changes to it based on PWLE feedback.
**14**	Identify roles, responsibilities, and concrete tasks for PWLE at each stage of the research project, including developing and implementing the research, understanding the data, and reporting on the findings.
**15**	Budget appropriately for engagement to ensure that PWLE are promptly and regularly paid a fair hourly rate for all contributions.
**16**	Develop an appropriate engagement format for your project that considers PWLE availability. This might include PWLE attendance or co‐facilitation of advisory group meetings or steering committee meetings.
**17**	When appropriate, learn about culturally inclusive engagement practices and how to apply them.
**18**	Learn about trauma‐informed engagement practices and look for ways to incorporate them.
**Onboarding people with lived experience**	
**19**	Engage more than one PWLE on each project to ensure that diverse experiences are heard and to provide opportunities for them to support each other.
**20**	Hold a group onboarding meeting to introduce PWLE to each other and to the research team.
**21**	Take the time to establish friendly rapport and to build relationships with PWLE.
**22**	Partner with PWLE to develop a document that sets out the roles, tasks, time commitment, and freedom to withdraw for each member of the team.
**23**	Provide opportunities to develop rapport and inquire about accessibility needs, preferred methods of communication, goals, and payment preferences (e.g. frequency and method of payment).
**24**	Check in with PWLE about the language and pronouns they want used to refer to them, respect their preferences, and be open to more than one option.
**25**	Inform PWLE about their privacy rights and the importance of confidentiality.
**Working with people with lived experience**	
**26**	Provide ongoing training, support, and mentorship to PWLE in the topic, the research, engagement skills, and any other skills necessary for the role.
**27**	Acknowledge and address unequal power between researchers and PWLE, for example, through training and by fostering a culture of respect.
**28**	Encourage the researchers and PWLE to reflect on and openly discuss their biases, for example, how their assumptions about mental health may shape the research project.
**29**	Be aware of historical and present inequities, stigma, and social and cultural barriers in research affecting the study population and work together to promote inclusivity.
**30**	While being flexible about scheduling and absences, check in with PWLE who are absent multiple times and support them in re‐engaging if they wish.
**31**	Be flexible with meeting times, geographic location, and personal factors that might impact engagement.
**32**	Create agendas and presentations that are clear, accessible, visual, jargon‐free, and stigma‐free.
**33**	Offer PWLE the option of having pre‐brief meetings, de‐brief meetings, or individual check‐ins as needed.
**34**	Ensure that PWLE have the time and opportunities to contribute in ways that are meaningful to them.
**35**	Be open to PWLE suggestions and be willing to make changes to the project, even if they do not align with your original vision for the project.
**36**	Maintain awareness that certain topics could be triggering and provide options in advance, such as skipping a meeting, leaving a meeting early, or attending an individual debrief session.
**37**	Regularly tell PWLE what feedback you have incorporated, and what you have not; provide a rationale for anything you were not able to incorporate.
**38**	Provide an accessible, user‐friendly way to track PWLE hours worked, recognizing that some PWLE may need support for this task; ensure prompt payment in cash, cash equivalent, or another PWLE‐preferred format.
**39**	At project closure, have a final PWLE meeting highlighting their contributions, explaining the next steps, and providing information about other engagement opportunities if available.
**Evaluating and reporting on PWLE engagement**	
**40**	Make a plan to evaluate the PWLE engagement process and report on it (e.g., PWLE experiences, impacts of engagement on the project).
**41**	Determine whether the extent of PWLE contributions make them eligible for manuscript coauthorship, invite them to coauthor the manuscript if appropriate, and support them in this role.
**42**	On manuscripts, acknowledge any PWLE who are not co‐authors, either by name or anonymously as per their preference.
**43**	When publishing, report accurately on the role of PWLE in your project.
**44**	Implement opportunities for PWLE to provide feedback on their overall experience with research engagement.

*Note:* This table contains the final items only. For the fully formatted Best Practice Guidelines, with explanatory text, please see the Appendix or https://www.camh.ca/en/professionals/professionals--projects/engaging-people-with-lived-experience-in-research.

## 4 Discussion

This modified virtual Delphi consensus study sought to develop best practice guidelines for the engagement of PWLE in mental health and substance use health research through the expert evaluations of PWLE and researchers. While most draft items were endorsed at high levels of importance, some items did not reach consensus, and most items were revised over the course of the project to better reflect the perspectives of participants. The resulting guidelines have been developed directly with PWLE and research teams to guide research teams in the engagement of PWLE in authentic, meaningful, and productive manners. The engagement of PWLE both as expert panelists and in a PWLE advisory group, i.e., using both deliberative and consultative engagement [44], contributed to optimizing the quality of the data collected.

These guidelines are both unique and consistent with the guidelines set out for engagement in health research as a whole. The PEIR conceptual framework [12, 13], the UK Public Involvement Standards [14], and the recommendations provided by organizations like PCORI [15], Wellcome Trust [15] and other bodies all tend to key components of engagement such as the importance of building relationships, showing respect, providing opportunities for authentic contribution throughout the research cycle, flexibility, and evaluating engagement. These aspects of all guidelines, then, appear to be key principles that apply to engagement across health research, providing some level of generalizability. The current guideline was developed based on an empirical research process involving PWLE and researchers, which is not always the case for all guidance documents. The process led to an extensive range of practical, actionable recommendations that apply to the full scope of the research engagement process, from early reflections to concrete practices to evaluation. This guideline is also unique for its focus on mental health and substance use health.

While engagement takes place across health sciences research, some specific considerations should be kept in mind in the mental health and substance use health sphere [2]. Given issues such as past and current inequities, the importance of hope, high levels of stigma and trauma, and the potential interaction between mental health symptoms and engagement practices, mental health and substance use health should be taken into specific consideration in PWLE engagement practices. This is evidenced in a number of items in the best practice guidelines proposed. Multiple items address issues related to inequities, stigma, and cultural inclusivity, as well as the importance of flexibility to work with PWLE to the extent and in the ways that they are able. One item focuses on the language used to refer to PWLE, which is often different from patient‐oriented research terminology, has been debated, and is not subject to consensus [45]. Items on trauma‐informed practice and triggers are important in the mental health sphere, given the extremely high rates of trauma among this population [46]. Family involvement also has specific considerations in the mental health and substance use health sector [47]. Many of these concepts have not been covered by previous engagement guideline documents from outside of the mental health sector [48, 49] [20]. These items therefore reflect some of the specificity of the mental health and substance use health sector.

Despite the development of the items with mental health and substance use health specifically in mind, some items might be hypothesized to be relevant outside of this field of research. For example, reflecting on one's motivations for engagement, building strong relationships, providing fair payment, and ensuring that institutional supports are available would likely benefit engagement initiatives across the health sciences [50]. Even some of the mental health‐specific items might have broader generalizability. Notably, mental health conditions are common enough in the general population that any engagement group is likely to include people with mental health challenges within it [51]. Conducting trauma‐informed engagement is particularly important in mental health, where some 15% of people with severe mental illness also meet diagnostic criteria for a comorbid posttraumatic stress disorder [46]. However, this might also be important in other health populations, since 90% of the general population has been exposed to a traumatic event at some point in their lives [52]. Engagement practices that are sensitive to mental health and trauma should therefore be the norm. Researchers in other domains of health might consider reviewing these recommendations, together with PWLE, to identify areas of overlap and utility.

Family engagement is often considered separately from the engagement of people with personal lived experience in mental health and substance use health research, coming with its own specific challenges, yet is under‐studied [6, 47]. Our advisory group, which was comprised of individuals with personal lived experience of mental health and/or substance use conditions and family members or caregivers of those with such conditions, felt strongly that families have their own lived experiences. Therefore, they recommended that the Delphi items refer to them collectively as one group (i.e., PWLE). However, based on participant comments in Round 1, we added one item specific to family members, which was immediately voted out in Round 2. This demonstrated that expert panelists agreed with our advisory group members in this regard – that families should be referred to together with people with personal lived experience and that they should be engaged following the same set of best practice guidelines that apply to people with personal lived experience. However, some may disagree with this merger of the two groups. One item was retained due to a discrepant rating between family members and the sample as a whole (i.e., *Engage two or more PLWE*), but the rest of the items showed consistency, further demonstrating that the two groups agreed. Any potential differences between engagement practices for people with personal lived experience and family/caregiver lived experience is a relevant area for future research.

The dissemination and uptake of best practice guidelines is consistently a challenge in the health sciences [53]. It is important that the current results be disseminated to research teams conducting PWLE engagement in the mental health and/or substance research sector, as well as to the PWLE engaged who might not have access to academic subscriptions. The current article, published in open access form, presents the final items for equitable access to research teams and PWLE alike [54]. In addition, a fully formatted best practice guideline, with an accompanying narrative, is provided in the public domain and is readily available in the Appendix by contacting the authors. This article and the supplementary best practice guideline will be shared publically amongst interested expert panelists and broadly via the social media of the investigators and knowledge users. Additional opportunities, such as presentations, trainings, and everyday language summaries will be explored. The guidelines will further be made available amongst engagement tools and resources on organizational websites. Researchers conducting PWLE engagement are encouraged to follow the guidelines, provide input on their utility for ongoing improvement, and support the further dissemination of them in their networks. They are further encouraged to evaluate the impact of engagement on the basis of engagement quality, which is an identified evidence gap in the engagement literature [31].

Strengths and limitations should be kept in mind. This was a lived experience‐engaged process, resulting in best practice guidelines that were co‐designed with PWLE. Researchers and PWLE including family members were all represented. The guidelines emerge from participants across racial/ethnic backgrounds, ages, and Canadian provinces. They also emerge from researchers working across child, youth, adult, and geriatric populations. However, geographic diversity was limited; likewise, the majority of participants were women, leaving uncertainty with regard to the representation of men and people with transgender or nonbinary identities. We did not ask participants whether they were engaged on the basis of mental health versus substance use health conditions, which limits the differentiation of the findings in the interpretation of the findings. The research participants were largely young professionals, with few late‐career scientists represented. While some subgroup analyses were conducted from a diversity perspective, this study did not produce culture‐specific guidelines for Indigenous populations, 2SLGBTQIA+ populations, immigrant populations, other sub‐populations, or the intersectionalities between them. There was no in‐person meeting of the expert panels, which might have produced rich conversations that could have affected the final results.

## 5 Conclusions

There are many advantages to engaging PWLE in mental health and/or substance use health research, providing benefits for those engaged, research teams, and the research itself. However, PWLE engagement can be challenging and sometimes requires thinking in new ways. These co‐developed best practice guidelines provide recommendations that research teams can follow to engage PWLE meaningfully, from the initial intention to engage through to the end‐of‐grant evaluation process. By following practices identified by research teams and PWLE, it may be possible to avoid tokenistic engagement by conducting high‐quality engagement activities in which PWLE contributions are truly appreciated and judiciously applied, ultimately having positive impacts on all parties involved, as well as on the research itself.

## Author Contributions

**Lisa D. Hawke:** conceptualization, investigation, funding acquisition, writing–original draft, methodology, validation, formal analysis, project administration, supervision, resources, data curation. **Wuraola Dada‐Phillips:** investigation, writing–original draft, formal analysis. **Hajar Seiyad:** investigation, methodology, writing–review and editing, formal analysis. **Josh Orson:** investigation, methodology, writing–review and editing, formal analysis. **Lianne Goldsmith:** investigation, methodology, writing–review and editing, formal analysis. **Susan Conway:** investigation, methodology, writing–review and editing, formal analysis. **Adam Jordan:** investigation, methodology, writing–review and editing, formal analysis. **Natasha Y. Sheikhan:** investigation, methodology, writing–review and editing. **Melissa Hiebert:** investigation, methodology, writing–review and editing. **Sean Kidd:** investigation, writing–review and editing, methodology. **Kerry Kuluski:** investigation, methodology, writing–review and editing.

## Conflicts of Interest

The authors declare no conflicts of interest.

## Supporting information

Supporting information.

## Data Availability

The data that support the findings of this study are available from the corresponding author upon reasonable request and with research ethics board approval.
