# Supplementary material for: Best Practices Guidelines for the Engagement of People With Lived Experience and Family Members in Mental Health and Substance Use Health Research: A Modified Delphi Consensus Study
Source: Health Expect. 2025 Jan 20;28(1):e70152. doi: 10.1111/hex.70152 (PMC11745228; doi:10.1111/hex.70152)
Supplement: Supplementary file 1 — Supporting information. [file HEX-28-e70152-s001.pdf]

camh
mental health is health

Best Practice Guidelines

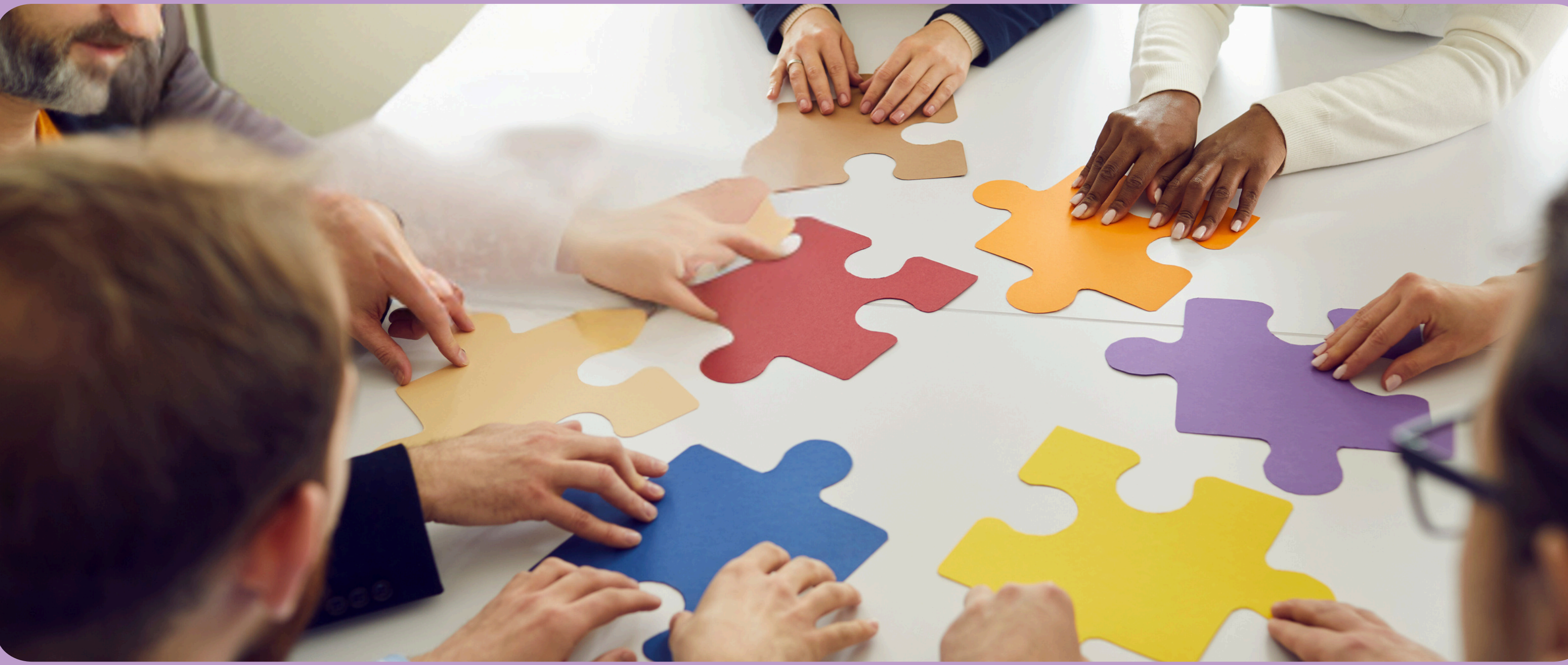

For the engagement of people with lived experience in mental health/substance use health research

www.camh.ca

This document was produced by a research team led out of the Centre for Addiction and Mental Health. The project was funded by the Canadian Institutes of Health Research.

Suggested citation: Lisa D. Hawke, Wuraola Dada-Phillips, Hajar Seiyad, Josh Orson, Lilian Goldsmith, Susan Conway, Adam Jordan, Natasha Y. Sheikhan, Melissa Hiebert, Sean Kidd, Kerry Kuluski. *Best Practice Guidelines for the Engagement of People with Lived Experience in Mental Health and Substance Use Health Research*. Centre for Addiction and Mental Health, Toronto, Canada. 2024

For more information about these guidelines or to submit feedback, contact

Lisa D. Hawke
Scientist, Centre for Addiction and Mental Health
CAMH Scientific Lead, Lived Experience and Family Engagement in Research
Assistant Professor, University of Toronto, Department of Psychiatry
60 White Squirrel Way, 316, Toronto, Ontario M6J 1H4
lisa.hawke@camh.ca

camh

CIHR IRSC
Canadian Institutes of Health Research
Instituts de recherche en santé du Canada

Contents

Introduction 1

Reflecting on underlying values 2

Planning to engage people with lived experience 3

Onboarding people with lived experience 5

Working with people with lived experience 6

Evaluating and reporting on engagement 9

Conclusions 10

Introduction

Engaging people with lived experience (PWLE) of mental health and substance use challenges and their family members in research is a growing priority for researchers, institutions, funding bodies, and PWLE themselves. However, historically, engagement has sometimes been limited by challenges such as tokenism, unequal power dynamics, and insufficient support for the PWLE engaged in the research. The *Best Practice Guidelines* provides recommendations for the engagement of PWLE in mental health and substance use health research. We use PWLE to include family members, who have their own lived experience.

These *Guidelines* emerged from a three-round modified virtual Delphi study conducted with researchers, research engagement staff, and PWLE, including families. The study was conducted in 2024 with 61 participants (35 PWLE including family members, 26 researchers). Participants rated the importance and clarity of each of the items and provided open-ended responses to help shape the final *Guidelines*. After each round, items were deleted, added, and reworded for clarity.

PWLE advisors were part of the research team that conducted the study. They were actively involved in planning the study, reviewing each round of data, developing and rewording the recommendations, and reporting on the results. Both the study process and the final *Guidelines* thereby reflect the priorities and perspectives of PWLE.

These *Best Practice Guidelines* cover key aspects of engaging PWLE in mental health and substance use health research. They are designed to support research teams in integrating PWLE in all stages of research. Research teams are encouraged to read through the *Guidelines*, see how they apply, and use them to support their PLWE engagement activities. While it is possible that some items do not apply to certain situations, they are designed to be as broadly applicable as possible. We encourage research teams to consider how the items might apply to their situation and use them accordingly. The recommendations are grouped into 5 sections taking readers through the stages of a research project. Each section starts with a brief introduction, followed by specific recommendations.

Our team invites your feedback on these *Best Practices Guidelines,* including their clarity and applicability, as well as any information that might guide future iterations. Contact information is available on the copyright page at the beginning of the document.

Reflecting on underlying values

This section includes recommendations focusing on principles that research teams are encouraged to examine before beginning to engage PLWE in research. By taking the time to reflect upon these aspects of the engagement initiative, a research team will be setting the stage for authentic, meaningful, and collaborative engagement alliance. We encourage research teams to examine each of these findings when first considering engaging PWLE in their work, to revisit these recommendations regularly, and to build upon them with their own experiences as their work progresses.

Recommendation 1

Consider why you want to engage PWLE and how their expertise can be included in your project.

Recommendation 2

Recognize that lived experience and research experience are different but equally valuable. Both can inform a research project.

Recommendation 3

Acknowledge that PWLE are experts who can inform research, make the research more relevant, and improve its quality.

Recommendation 4

Acknowledge that PWLE can identify factors influencing the research questions that you might have otherwise overlooked.

Recommendation 5

Recognize that everyone’s experience is unique and that one PWLE cannot represent all people.

Recommendation 6

Appreciate the unique insights (about age, gender, mental health, etc.) that PWLE provide.

Planning to engage people with lived experience

These recommendations focus on planning steps that research teams can work through before beginning to engage PWLE. They require examining the objectives of the research project at hand, as well as any opportunities and constraints that may affect the research team and the research project. Some recommendations require a commitment to learning and addressing potentially uncomfortable topics, such as inequities, stigma, and unequal power. Others focus on concrete planning steps that will help teams develop an appropriate PWLE engagement process, such as attention to critical aspects like representativeness and leveraging PWLE perspectives to maximize the research potential, alongside budgeting, fair payment, accessibility, and meeting structures.

Recommendation 7

Plan to start engaging PWLE as early as possible in the research project.

Recommendation 8

Ensure that the team's scientists, trainees, and research staff have appropriate training on how to engage meaningfully with PWLE.

Recommendation 9

Identify what health conditions (e.g., mental health, physical health) or other characteristics (e.g., age, gender, ethnicity) you want the PWLE to have, based on the goals of the study.

Recommendation 10

Consider what populations you are intentionally or unintentionally excluding from your research and from your engagement initiative, why, and how that impacts your project.

Recommendation 11

Explore where in the broader community you can find diverse PWLE to engage.

Recommendation 12

Develop materials to recruit PWLE who are representative of your priority population, with a clear indication of the role and commitment.

Recommendation 13

Plan to be flexible in the project and make changes to it based on PWLE feedback.

Recommendation 14

Identify roles, responsibilities, and concrete tasks for PWLE at each stage of the research project, including developing and implementing the research, understanding the data, and reporting on the findings.

Recommendation 15

Budget appropriately for engagement to ensure that PWLE are promptly and regularly paid a fair hourly rate for all contributions.

Recommendation 16

Develop an appropriate engagement format for your project that considers PWLE availability. This might include PWLE attendance or co-facilitation of advisory group meetings or steering committee meetings.

Recommendation 17

When appropriate, learn about culturally inclusive engagement practices and how to apply them.

Recommendation 18

Learn about trauma-informed engagement practices and look for ways to incorporate them.

Onboarding people with lived experience

Onboarding PWLE to a research team is a key step that prepares the team for the rest of the engagement experience. An attentive process of introducing the PWLE to the research team sets the stage and establishes a sustainable environment for effective engagement down the road. These recommendations therefore include steps that we encourage research teams to take when first launching PWLE engagement.

Recommendation 19

Engage more than one PWLE on each project to ensure that diverse experiences are heard and to provide opportunities for them to support each other.

Recommendation 20

Hold a group onboarding meeting to introduce PWLE to each other and to the research team.

Recommendation 21

Take the time to establish friendly rapport and to build relationships with PWLE.

Recommendation 22

Partner with PWLE to develop a document that sets out the roles, tasks, time commitment, and freedom to withdraw for each member of the team.

Recommendation 23

Provide opportunities to develop rapport and inquire about accessibility needs, preferred methods of communication, goals, and payment preferences (e.g., frequency and method of payment).

Recommendation 24

Check in with PWLE about the language and pronouns they want used to refer to them, respect their preferences, and be open to more than one option.

Recommendation 25

Inform PWLE about their privacy rights and the importance of confidentiality.

Working with people with lived experience

This section focuses on the way research teams engage PWLE during the actual research process. With strong initial reflections, planning, and onboarding processes in place, research teams may be ready to move collaboratively into the project operations. This is where research teams will begin to get the concrete feedback they are looking for to guide many aspects of the research project, from defining the research questions through to knowledge translation processes. In doing so, it is important to get ongoing feedback from PWLE on the process to make sure the work is reciprocal and mutually beneficial. Ongoing reflection, open discussion, and consistent flexibility—underpinned by honesty, transparency and respect—are key to effective engagement work with PWLE.

Recommendation 26

Provide ongoing training, support, and mentorship to PWLE in the topic, the research, engagement skills, and any other skills necessary for the role.

Recommendation 27

Acknowledge and address unequal power between researchers and PWLE, for example, through training and by fostering a culture of respect.

Recommendation 28

Encourage the researchers and PWLE to reflect on and openly discuss their biases, for example, how their assumptions about mental health may shape the research project.

Recommendation 29

Be aware of historical and present inequities, stigma, and social and cultural barriers in research affecting the study population and work together to promote inclusivity.

Recommendation 30

While being flexible about scheduling and absences, check in with PWLE who are absent multiple times and support them in re-engaging if they wish.

Recommendation 31

Be flexible with meeting times, geographic location, and personal factors that might impact engagement.

Recommendation 32

Create agendas and presentations that are clear, accessible, visual, jargon free, and stigma free.

Recommendation 33

Offer PWLE the option of having pre-brief meetings, de-brief meetings, or individual check-ins as needed.

Recommendation 34

Ensure that PWLE have the time and opportunities to contribute in ways that are meaningful to them.

Recommendation 35

Be open to PWLE suggestions and be willing to make changes to the project, even if they do not align with your original vision for the project.

Recommendation 36

Maintain awareness that certain topics could be triggering and provide options in advance, such as skipping a meeting, leaving a meeting early, or attending an individual debrief session.

Recommendation 37

Regularly tell PWLE what feedback you have incorporated, and what you have not; provide a rationale for anything you were not able to incorporate.

Recommendation 38

Provide an accessible, user-friendly way to track PWLE hours worked, recognizing that some PWLE may need support for this task; ensure prompt payment in cash, cash equivalent, or another PWLE-preferred format.

Recommendation 39

At project closure, have a final PWLE meeting highlighting their contributions, explaining the next steps, and providing information about other engagement opportunities if available.

Evaluating and reporting on lived experience engagement

Once your research project is nearing its end, there are still PWLE engagement processes to keep in mind, such as reporting and evaluation. While evaluation is mentioned at the end of these *Guidelines* as part of the reporting process, evaluation should be planned from the outset so that you are ready to conclude your project on a strong footing. When authoring publications and other reports about your research, it is important to remember the substantial contributions that PWLE have made all the way through the project and to recognize those contributions in appropriate manners. The PWLE you worked with are colleagues whose contributions have helped to make the research project all that it has been.

Recommendation 40

Make a plan to evaluate the PWLE engagement process and report on it (e.g., PWLE experiences, impacts of engagement on the project).

Recommendation 41

Determine whether the extent of PWLE contributions make them eligible for manuscript co-authorship, invite them to co-author the manuscript if appropriate, and support them in this role.

Recommendation 42

On manuscripts, acknowledge any PWLE who are not co-authors, either by name or anonymously as per their preference.

Recommendation 43

When publishing, report accurately on the role of PWLE in your project.

Recommendation 44

Implement opportunities for PWLE to provide feedback on their overall experience with research engagement.

Conclusions

PWLE make substantial contributions to mental health and substance use health research. We hope that these *Best Practice Guidelines* will help research teams work productively and authentically with PWLE. In doing so, we are confident that they will be able to improve the quality and relevance of their research. We call on research teams to apply these Guidelines to support their engagement and, if they're willing, report back to us on their applicability and utility.

The development of these *Best Practice Guidelines* was an extensive process involving multiple PWLE, with efforts to be comprehensive in content coverage. However, it is possible that some important aspects of engagement may have been missed. When working with PWLE, it is important to remain thoughtful about the planning, the process, and the outcomes, with constant flexibility and openness to new ideas. We encourage research teams to observe and document effective PWLE engagement practices that work for them. Likewise, we encourage teams to identify challenging practices, to reflect on mitigating strategies, and share these experiences with the PWLE engagement research community so that we can all continue to improve our work together.

These *Guidelines* were developed with specific attention to issues related to mental health and substance use. This is reflected in items about issues like stigma, trauma, and inclusivity, among others. While it is hoped that following these *Guidelines* will help create a safe and inclusive space for PWLE in the mental health and substance use health sector, it is possible that some of the recommendations may also apply outside of this area of research. Research teams and PWLE working outside of mental health and substance use health research might consider reflecting on these *Guidelines* and considering how they might apply to their area of work. Adaptations for other areas of research, as well as for specific subpopulations, may be an area for future work.

Lastly, we sincerely thank all of the expert panel members and the research team members who contributed to the development of these *Best Practice Guidelines*. Their commitment to working together to improve the process of PWLE engagement is recognized and appreciated as a common goal that we all share. As we continue on this path, each and every person who contributed to this work is leading the way to improve PWLE engagement in research, and to thereby improve the quality of research, the evidence base, and ultimately our health care experiences and practices.

www.camh.ca
